# Comparative efficacy of non-electric cooling techniques to reduce nutrient solution temperature for the sustainable cultivation of summer vegetables in open-air hydroponics

**DOI:** 10.3389/fpls.2024.1340641

**Published:** 2024-03-01

**Authors:** Muhammad Mohsin Nisar, Rashid Mahmood, Salman Tayyab, Moazzam Anees, Faisal Nadeem, Sadia Bibi, Faiza Waseem, Nazir Ahmed, Jing Li, Zhao Song

**Affiliations:** ^1^ Key Laboratory for New Technology Research of Vegetables, Vegetable Research Institute, Guangdong Academy of Agricultural Science, Guangzhou, China; ^2^ Department of Horticulture, University of the Punjab, Lahore, Pakistan; ^3^ Department of Soil Science, University of the Punjab, Lahore, Pakistan; ^4^ Institute of Soil and Environmental Sciences, University of Agriculture Faisalabad, Faisalabad, Pakistan; ^5^ College of Horticulture and Landscape Architecture, Zhongkai University of Agriculture and Engineering, Guangzhou, Guangdong, China

**Keywords:** hydroponics, nutrient solution temperature, PVC grow pipes, jute fabric, open air system

## Abstract

The cultivation of summer vegetables in open-air nutrient film technique (NFT) hydroponics is limited due to the elevated nutrient solution temperature (NST). In this regard, non-electric evaporative-cooling techniques were explored to maintain NST in open-air NFT hydroponics. Four cooling setups were employed by wrapping polyvinyl chloride (PVC) grow pipes with one and two layers of either wet or dry jute fabrics and attaching them with coiled aluminum pipe buried inside a) wet sand-filled brick tunnels (Cooling Setup I), b) two inverted and vertically stacked earthen pots (Cooling Setup II), c) two inverted and vertically stacked earthen pots externally wrapped with wet jute fabric (Wrapped Cooling Setup II), and d) an earthen pitcher wrapped with wet jute fabric (Cooling Setup III). Wrapping grow pipes with two layers of wet jute fabric reduced NST by 5°C as compared to exposed (naked) grow pipes. The double-layer jute fabric-wrapped grow pipes produced 182% more reduction in NST in comparison to single layer-wrapped grow pipes. Additionally, the installation of Wrapped Cooling Setup II and Cooling Setup III outperformed Cooling Setup I and Cooling Setup II through NST reduction of approximately 4°C in comparison to control. Interestingly, Cooling Setup III showed its effectiveness through NST reductions of 193%, 88%, and 23% during 11 a.m.–12 p.m. as compared to Cooling Setup I, Cooling Setup II, and Wrapped Cooling Setup II, respectively. In contrast, Wrapped Cooling Setup II caused NST reductions of 168%, 191%, and 18% during 2–3 p.m. in comparison to Cooling Setup I, Cooling Setup II, and Cooling Setup III, respectively. Thus, the double-layer jute fabric-wrapped grow pipes linked with Wrapped Cooling Setup II can ensure summer vegetable cultivation in open-air NFT hydroponics as indicated by the survival of five out of 12 vegetable plants till harvest by maintaining NST between 26°C and 28°C.

## 1 Introduction

Hydroponic systems include deep water culture, ebb and flow, drip system, aeroponics, wick system, vertical farming system, and nutrient film technique (NFT) (Roberto, 2000). NFT hydroponic cultures have benefits over soil cultures such as efficient utilization of resources (water, nutrients, and space), better control of pests and diseases, faster growth, and higher yields. However, high initial cost, power dependency, system failure risks, limited crop compatibility, dependence on technical expertise, intensive monitoring, and system maintenance are the common limitations of NFT hydroponics (Swain et al., 2021). Given the rate of population increase and subsequent urbanization (Velazquez-Gonzalez et al., 2022), the food demand of the growing world population can only be met through the strengthening of alternate plant growth mediums. NFT hydroponics can ensure higher yield year-round through the accommodation of increased plant population of multiple crops by the effective utilization of its horizontal as well as vertical surface area compared to traditional farming systems. Moreover, the minimal escape of pesticides and fertilizers in NFT hydroponic cultures ensures lesser environmental contamination (van Delden et al., 2021).

The nutrient solution, either pumped from a reservoir tank or kept stagnant in aerated containers, remains in direct contact with plant roots in NFT hydroponics. Simple as well as modified NFT hydroponics systems are used for the commercial cultivation of vegetable crops, like lettuce, tomato, cucumber, herbs, and other green vegetables (Stephanie, 2018; Sela Saldinger et al., 2023). Various attributes of nutrient solution, e.g., nutrient concentration, dissolved oxygen, temperature, pH, and electrical conductivity, are of critical importance and need to be monitored regularly to ensure healthy crop production (Jones, 2004). Among these attributes, temperature is an important factor determining plant survival through root and shoot development in NFT hydroponics. Any temperature fluctuations, from an optimum range, subject plants to adverse stress through changes in nutrient solubility, uptake, and proportions of dissolved oxygen in the rhizosphere (Jones, 2004; Ylivainio and Peltovuori, 2012; Xia et al., 2021). The reduction of dissolved oxygen under high temperatures can prove drastic for plant growth and development. Vegetable plant species are reported to have differences in their desired optimum temperature. For instance, the optimum root zone temperature is reported to be 25°C for tomato (Tindall et al., 1990), whereas cucumber produces a higher yield at 22°C (Al-Rawahy et al., 2018). On an overall basis, 26.7°C (80°F) is the nutrient solution temperature where maximum absorption of nutrient elements is reported; however, 25°C (77°F) is considered to be the ideal temperature for maximum root and shoot growth (Jones, 2004). Nonetheless, the seasonal air temperature determines the extent of temperature fluctuations happening in the nutrient solution of the NFT system. In winter, maintaining the temperature at 28°C and 20°C proved optimum for the production of spinach and cucumber, respectively (Nxawe et al., 2009; Yan et al., 2013).

The cultivation of in-season summer vegetable crops in an open-air hydroponics can save the cost of house enclosure and air conditioning for temperature maintenance. However, maintaining nutrient solution temperature lower than the external air temperature is a challenge in an open-air hydroponic system. Thus, it becomes critically important to explore cooling systems for nutrient solutions in NFT hydroponics. In the view of high installation costs of NFT hydroponic systems, the effectiveness of non-electric techniques (jute fabric wrapping and aluminum coil buried in brick walls, earthen pots, and pitchers), to keep the nutrient solution temperature at an optimum range, has never been reported. The study aims to explore the non-electric techniques focusing, specifically, on passive evaporation to maintain nutrient solution temperature in NFT hydroponics for the cultivation of summer vegetables. This investigation is motivated by the desire to achieve nutrient solution temperature reductions without the association of expensive (electric energy-driven) methods.

## 2 Materials and methods

**Figure 1:**
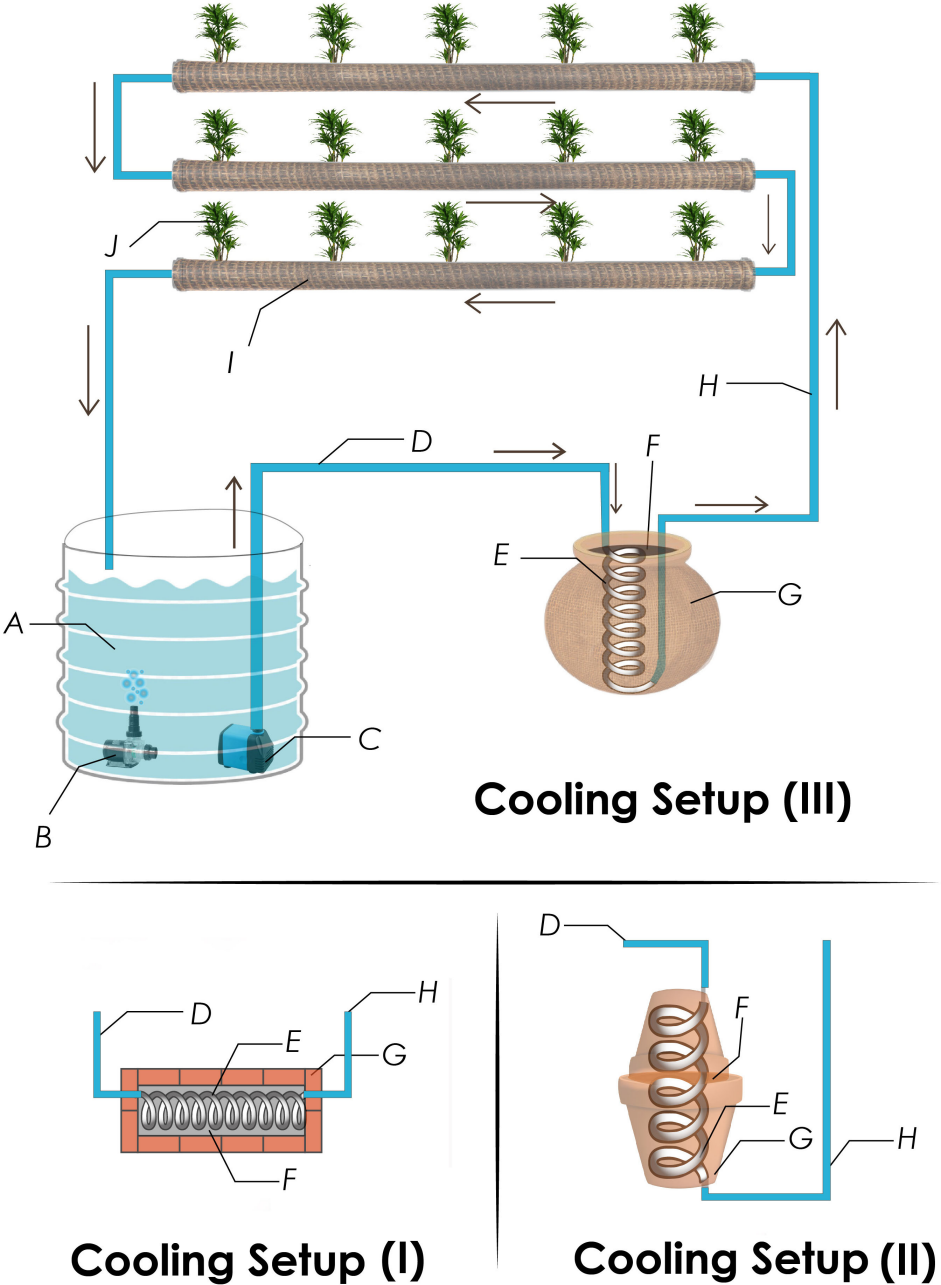
Schematic diagram of nutrient film technique (NFT) mounted with one of the three solution cooling setups. Various parts of the NFT include nutrient solution tank (A), aeration pump (B), and nutrient circulating pump (C), which pumps nutrients solution through input pipe (D) of a cooling setup to the inner aluminum pipe coil (E) that is buried in wet sand (F) to transfer heat to the external evaporative surface (G) of brick lining (Cooling Setup I), earthen pots (Cooling Setup II), or pitcher (Cooling Setup III). After that, the solution moves through output pipe (H) of a cooling setup to the jute-wrapped grow pipes (I) and ultimately back to the solution tank.

**Figure 2:**
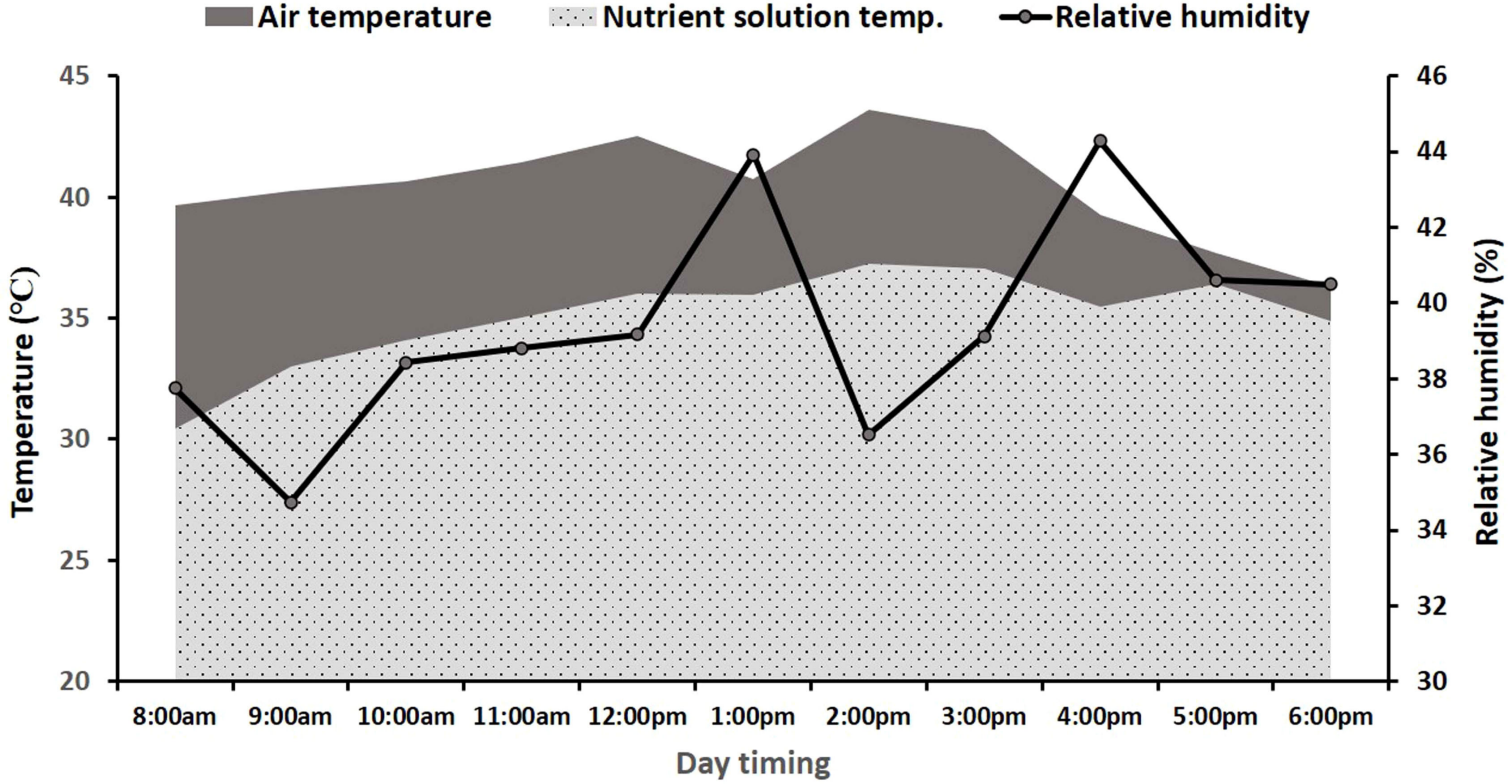
Variations with day timing in air temperature, relative humidity, and nutrient solution temperature in nutrient film technique (NFT) naked pipes (data are averages of 47 days from May 17, 2022, to July 2, 2022).

The NFT hydroponic setup was installed in the wire house of the Department of Soil Science, Faculty of Agricultural Sciences, University of the Punjab, Lahore, Pakistan. The setup comprised a nutrient solution tank, a submerged solution circulating pump, an aeration pump, connecting tubes, and polyvinyl chloride (PVC) pipes. Each PVC pipe had 4-m length and 10-cm diameter, with 10 planting holders acting as a grow pipe (**Figure 1**). The nutrient solution of NFT hydroponic system consisted of 2 mM NH_4_NO_3_, 0.25 mM KH_2_PO_4_, 0.75 mM K_2_SO_4_, 0.1 mM KCl, 2 mM CaCl_2_, 0.65 mM MgSO_4_, 0.2 mM Fe-EDTA, 1 × 10^−3^ mM MnSO_4_, 1 × 10^−3^ mM ZnSO_4_, 1 × 10^−4^ mM CuSO_4_, 5 × 10^−6^ mM (NH_4_)_6_Mo_7_O_24_, and 1 × 10^−3^ mM H_3_BO_3_ (Nadeem et al., 2018). The NFT hydroponics system was operated in open-air conditions during the summers (May 16 to July 2, 2022) under the daytime air and nutrient solution temperatures as described in **Figure 2**.

### 2.1 Experiment 1: jute fabric wrapping of grow pipes to reduce nutrient solution temperature

In this experiment, the investigations were focused on the utilization of jute fabric for wrapping PVC grow pipes as a tool to reduce nutrient solution temperature (NST). The experiment followed a completely randomized design (CRD) and considered three factors: the number of jute fabric layers used in wrapping (single-layer wrapping and double-layer wrapping), the condition of wrapped jute fabric (wet or dry), and the time of NST measurement (7 a.m.–10 a.m. and 11 a.m.–5 p.m.). The jute fabric in single-layer wrapping and double-layer wrapping was kept either dry or saturated with water once in 24 hours. The saturation proved to be effective in ensuring a continuous water supply within the fabric supporting a 24-hour evaporation cycle. The temperature variations in the nutrient solution were recorded at least twice during both designated time intervals (7 a.m.–10 a.m. and 11 a.m.–5 p.m.). These time intervals were chosen based on the elimination of overnight cooling of the nutrient solution by approximately 10 a.m. The experiment was conducted in three replications (a 4-m-long PVC grow pipe with 10 plant holders as one replicate). Each replicate pipe was connected to a separate solution tank containing a solution circulating pump and an aeration nozzle. Cucumber seedlings at four leaf stages were transplanted to grow pipes, and the setup was run for 15 days (May 17, 2022, to May 31, 2022). During the experimental period, the wilted plants were replaced on the same day they were wilted.

Nutrient solution temperature, air temperature, and relative humidity were recorded four to five times during the day using a hygrometer (HTC-2; Walmart Canada, Mississauga, ON, Canada) equipped with an internal and cable-mounted external temperature sensor. The decrease in nutrient solution temperature in a jute fabric-wrapped grow pipe was recorded in comparison to that in a naked pipe (no wrapping) in two different time intervals (before 10 a.m. and from 11 a.m. to 5 p.m.), and the averages were calculated for each set.

### 2.2 Experiment 2: designing evaporative-cooling setups for the reduction of nutrient solution temperature

Four non-electric evaporative-cooling setups were developed (**Figure 1**) and installed in the circuit of nutrient solution individually. The details of these cooling setups are as follows.

#### 2.2.1 Cooling Setup I (treatment 1)

This setup was built on the floor by constructing a brick tunnel of 15-cm external height, 25-cm width, 75-cm length, and 4,875 cm^2^ of total external surface area for evaporation. The brick tunnel consisted of six sides in total: five sides (15 × 25 = 375 cm^2^, 15 × 25 = 375 cm^2^, 15 × 75 = 1,125 cm^2^, 15 × 75 = 1,125 cm^2^, and 25 × 75 = 1,875 cm^2^) exposed to air whereas the sixth side facing the ground and not contributing toward evaporative cooling. The total external surface area (4,875 cm^2^) was calculated by summing up the areas of all five air-exposed rectangular sides. An aluminum pipe of 0.79-cm diameter and 457-cm length was shaped into a coil with 6-cm diameter and 60-cm length and attached to the nutrient solution circuit such that the nutrient solution coming from the reservoir passed through the coil before entering the grow pipes. The coil was buried in the wet sand placed inside the brick tunnel structure. The roof of the tunnel was also covered with bricks (**Figure 1**). The whole structure and sand were placed in a shady area and saturated with water once in 24 hours.

#### 2.2.2 Cooling Setup II (treatment 2)

In this setup, the aluminum coil (similar to that used in Cooling Setup I) was buried in sand and placed inside two conical frustum-shaped earthen pots each of height (h) 35.0 cm, slant height (s) 37.5 cm, bottom radius (r) 4.0 cm, and top radius (R) 17.5 cm. These earthen pots were positioned such that one was inverted over the other, making the lateral surfaces of both the pots and bottom of the upper inverted pot available for evaporation (**Figure 1**). The evaporative surface areas of the lateral surface (LA) and bottom circular surface (BA) of the inverted pots were calculated by the following formulae (Weisstein, 2005);


LA=π×(R+r)×s

$$BA=\pi r^{2}$$

In this way, the total evaporative surface area (TA) of the setup was calculated to be 5,117.6 cm^2^ by using the following formula:


TA=2LA+BA

#### 2.2.3 Wrapped Cooling Setup II (treatment 3)

Cooling Setup II was modified by wrapping the external surface of earthen pots with wet jute fabric to facilitate evaporation.

#### 2.2.4 Cooling Setup III (treatment 4)

In this setup, the aluminum coil (similar to that used in Cooling Setup I) was immersed in the sand within a spherical earthen pitcher having a radius of 19.2 cm and a total surface area of 4,630.1 cm^2^. The evaporative surface area was calculated as 4,581.4 cm^2^ by subtracting the area of the circular mouth opening (28.3 cm^2^) and that of the ground touching the circular base (20.4 cm^2^) from the total surface area. The mouth opening of the pitcher was used for the entry and exit of the nutrient solution pipes. Therefore, the small amount of evaporation from this area was ignored.

All four cooling setups, including sand, were placed in a shaded area and saturated with water once in 24 hours. Through initial investigations, it was found that the periodic saturation effectively maintains sufficient water for evaporation throughout the structure until the subsequent saturation cycle. The NFT setup having none of the aforementioned cooling setups was taken as control. Each of the four cooling setups was attached with three grow pipes containing cucumber plants in holders and ran for 15 days from June 3, 2022, to June 18, 2022, in triplicate. Nutrient solution temperature, external air temperature, and relative humidity were recorded daily at four different time intervals, i.e., 8–9 a.m., 11 a.m.–12 p.m., 2–3 p.m., and 4–5 p.m., using a hygrometer as discussed in the case of experiment 1.

The experiment was conducted following the CRD with the type of cooling setups (Cooling Setup I, Cooling Setup II, Wrapped Cooling Setup II, and Cooling Setup III) and time intervals of nutrient solution temperature recording (8–9 a.m., 11 a.m.–12 p.m., 2–3 p.m., and 4–5 p.m.) as experimental factors. At a particular interval, the decrease in nutrient solution temperature due to a cooling setup was recorded in comparison to the control. The average values of 15 days were used in the statistical analysis.

### 2.3 Testing of the selected cooling techniques for the cultivation of summer vegetables

Keeping in view the results of experiment 1 and experiment 2, Wrapped Cooling Setup II was connected to double-layer wet jute fabric-wrapped grow pipes in the NFT system and tested for the cultivation of brinjal (*Solanum melongena*), okra (*Abelmoschus esculentus*), spinach (*Spinacia oleracea*), bitter gourd (*Momordica charantia*), coriander (*Coriandrum sativum*), sponge gourd (*Luffa aegyptiaca*), bottle gourd (*Lagenaria siceraria*), summer squash (*Cucurbita pepo*), pumpkin (*Cucurbita moschata*), cucumber (*Cucumis sativus*), and snake gourd (*Trichosanthes cucumerina*). An NFT system with naked grow pipes but none of the cooling setups in the circuit was considered as control. Three seedlings of each vegetable (**Table 1**), at four leaf stages, were shifted to grow pipes, and their survival duration was recorded for up to 2 months. The survival duration of the seedlings was recorded as the time taken by the seedlings from transplanting to permanent wilting. The confirmation of permanent wilting was performed by observing the plants in the grow holders for the subsequent 3 days. Two months after transplanting, the survived vegetable seedlings were carefully removed from grow holders and assessed for their growth in terms of root length, shoot length, number of leaves, shoot fresh weight, shoot dry weight, root fresh weight, and root dry weight. The number of leaves was counted, and the root and shoot lengths were measured using a meter rod. The shoot was separated from the root using a scissor and weighed through digital balance for shoot fresh weight. Any nutrient solution, adhering to the roots, was gently blotted with tissue paper for the measurement of root fresh weight. The dry weight was determined by keeping the shoot and root samples in an oven at 60°C till constant weight. For all the recorded growth parameters, the mean of the three seedlings of each vegetable was calculated and reported in **Table 2**. During the first week of the experiment, the temperature variations in the nutrient solutions were monitored at approximately 3 p.m. daily.

**Table 1:** Survival time of various vegetable seedlings cultivated in naked grow pipes and grow pipes wrapped with two layers of wet jute fabric and attached with wrapped Cooling Setup II.

Vegetables cultivated	Plant survival time	
Common name	Naked grow pipes	Wrapped grow pipes and wrapped Cooling Setup II in circuit
Brinjal (*Solanum melongena*)	7 days	Survived till harvest
Okra (*Abelmoschus esculentus*)	6 days	26 days
Spinach (*Spinacia oleracea*)	3 days	3 days
Bitter gourd (*Momordica charantia*)	7 days	Survived till harvest
Coriander (*Coriandrum sativum*)	2 days	2 days
Sponge gourd (*Luffa aegyptiaca*)	6 days	Survived till harvest
Bottle gourd (*Lagenaria siceraria*)	7 days	Survived till harvest
Summer squash (*Cucurbita pepo*)	3 days	4 days
Pumpkin (*Cucurbita moschata*)	4 days	6 days
Cucumber (*Cucumis sativus*)	6 days	Survived till harvest
Snake gourd (*Trichosanthes cucumerina*)	5 days	15 days

Survival time is an average of at least three plants of a species.

**Table 2:** Growth of vegetable seedlings survived till harvest in grow pipes wrapped with two layers of wet jute fabric and attached with wrapped Cooling Setup II.

Parameter	Brinjal	Bitter gourd	Sponge gourd	Bottle gourd	Cucumber
Root length (cm)	15.2 ± 1.4	10.2 ± 1.4	133.1 ± 12.05	7.2 ± 1.1	5.8 ± 0.6
Shoot length (cm)	36.6 ± 2.2	259.9 ± 13.5	995.7 ± 41.3	155.4 ± 2.9	75 ± 2.7
No. of leaves	8.4 ± 2.3	40.1 ± 4.2	56.7 ± 45.1	28.6 ± 13.5	25.6 ± 9.3
Shoot fresh weight (g)	4.9 ± 0.3	17.6 ± 3.9	138.7 ± 15.6	12.6 ± 3.3	13.3 ± 8.5
Root fresh weight (g)	0.5 ± 0.1	2.3 ± 0.3	32.7 ± 3.7	1.6 ± 3.7	2.2 ± 0.7
Shoot dry weight (g)	2.1 ± 0.2	5.6 ± 0.2	52.0 ± 2.7	3.1 ± 0.4	1.9 ± 0.1
Root dry weight (g)	0.1 ± 0.0	0.2 ± 0.0	3 ± 0.2	0.9 ± 0.1	0.1 ± 0.03

Values are mean ± SE.

### 2.4 Statistical analysis

For experiment 1, involving nutrient solution temperature reduction through wrapping of PVC grow pipes, the analysis of variance (ANOVA) was performed using Statistix (v. 8.1) to separate the main effects of three factors (number of jute layers wrapped, dry versus wet jute, and time of observation) as well as their interactions (**Table 3**). In addition, ANOVA was performed to find the effect of rates within each factor. The mean comparison of nutrient solution temperature reductions in wrapped grow pipes with respect to naked grow pipes was performed using Tukey’s honestly significant difference (HSD) test. For the comparison of four cooling systems in experiment 2, ANOVA was performed using Statistix (v. 8.1) to separate the main effects of factors (cooling system types and time of observation) as well as their interactions. In addition, ANOVA was also performed to find the effect of cooling systems on the nutrient solution temperature measured at different times of the day. The mean comparison of nutrient solution temperature reductions in grow pipes attached cooling setup with respect to control pipes (no cooling setup attachment) was made using Tukey’s HSD test.

**Table 3:** Analysis of variance for the impact of wrapping of nutrient solution carrying polyvinyl chloride (PVC) pipes with jute cloth on the solution temperature in nutrient film technique (NFT).

Source	F-ratio	p-Value
No. of jute layers wrapped (NJL)	11.59	0.001*
Dry vs. wet jute (DWJ)	75.30	0.000*
Time of observation (t)	16.13	0.000*
NJL × DWJ	0.47	0.497
DWJ × t	0.10	0.752
NJL × t	0.02	0.881
JW × t × NJL	0.48	0.489

*Significant at p ≤ 0.001. ns, non-significant at p< 0.05.

## 3 Results

Initially, the high nutrient solution temperature (up to 40°C) caused the death of all summer vegetable seedlings (**Table 1**) within 1 week (May 2022) of their transplantation in the NFT system. Various non-electric cooling techniques were implied and compared for their efficiency in reducing the nutrient solution temperature. During the study period, the external air temperature fluctuated from 30°C to 46°C (**Figure 2**). It caused the nutrient solution temperature to vary from 32°C to 37°C in naked grow pipes from 8 a.m. to 6 p.m. This range of nutrient solution temperature was 4.6°C, 3.3°C, 3.2°C, and 1.2°C lesser than the external air temperature recorded before 9 a.m., from 9 p.m. to 12 p.m., from 12 p.m. to 3 p.m., and 3 p.m. to 6 p.m., respectively. The nutrient solution temperature was minimal before 9 a.m. which increased by 3°C and 4.7°C up to 12 p.m. and 3 p.m., respectively, in naked grow pipes. However, the change in nutrient solution temperature from 3 p.m. to 6 p.m. was negligible (**Figure 2**). The relative humidity varied from 25% to 80% during the study period. It remained at approximately 30% to 40% from the start of May to the middle of June but fluctuated between 60% and 80% after June 15 due to pre-monsoon rains. However, the average daily relative humidity level during the study period was 35% to 44% (**Figure 2**).

### 3.1 Impact of jute fabric wrapping of grow pipes on nutrient solution temperature

**Figure 3:**
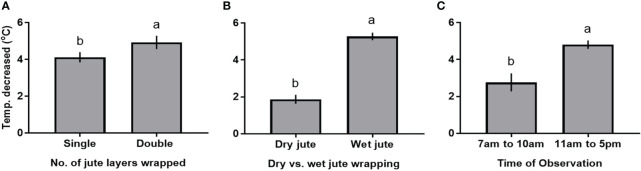
Main effects of number of jute layers wrapped over nutrient film technique (NFT) solution pipes **(A)**, dryness or wetness of the wrapped jute **(B)**, and time of the day **(C)** on the decrease in solution temperature in comparison to that in the naked pipe. Bars are means ± standard errors, and means sharing common letters do not differ significantly at p< 0.05.

The results of ANOVA highlighted the significant influence of grow pipe wrapping techniques to reduce nutrient solution temperature at different times of observations (**Table 3**). However, the interaction effects of the number of jute fabric layers wrapped, dry or wet jute fabric layer, and time of observation were non-significant at p<0.05 (**Table 3**). The nutrient solution temperature in jute fabric-wrapped grow pipes decreased by up to 5°C in comparison to naked grow pipes. Furthermore, the double-layer jute fabric wrapping proved approximately 20% more effective in reducing the solution temperature than the single-layer wrapping (**Figure 3A**, **Supplementary Table 1**). Consistently, the wet jute fabric wrapping proved to decrease nutrient solution temperature by 182% more than dry jute fabric wrapping (**Figure 3B**, **Supplementary Table 1**). On average, the decrease in nutrient solution temperature under double-layer jute fabric wrapping was 74% more than single-layer wrapping at all times of observation (**Supplementary Table 1**); however, the decrease was 39% more prominent during 11 a.m.–5 p.m. than 7 a.m.–10 a.m. (**Figure 3C**).

### 3.2 Impact of installation of various non-electric cooling setups on nutrient solution temperature

**Figure 4:**
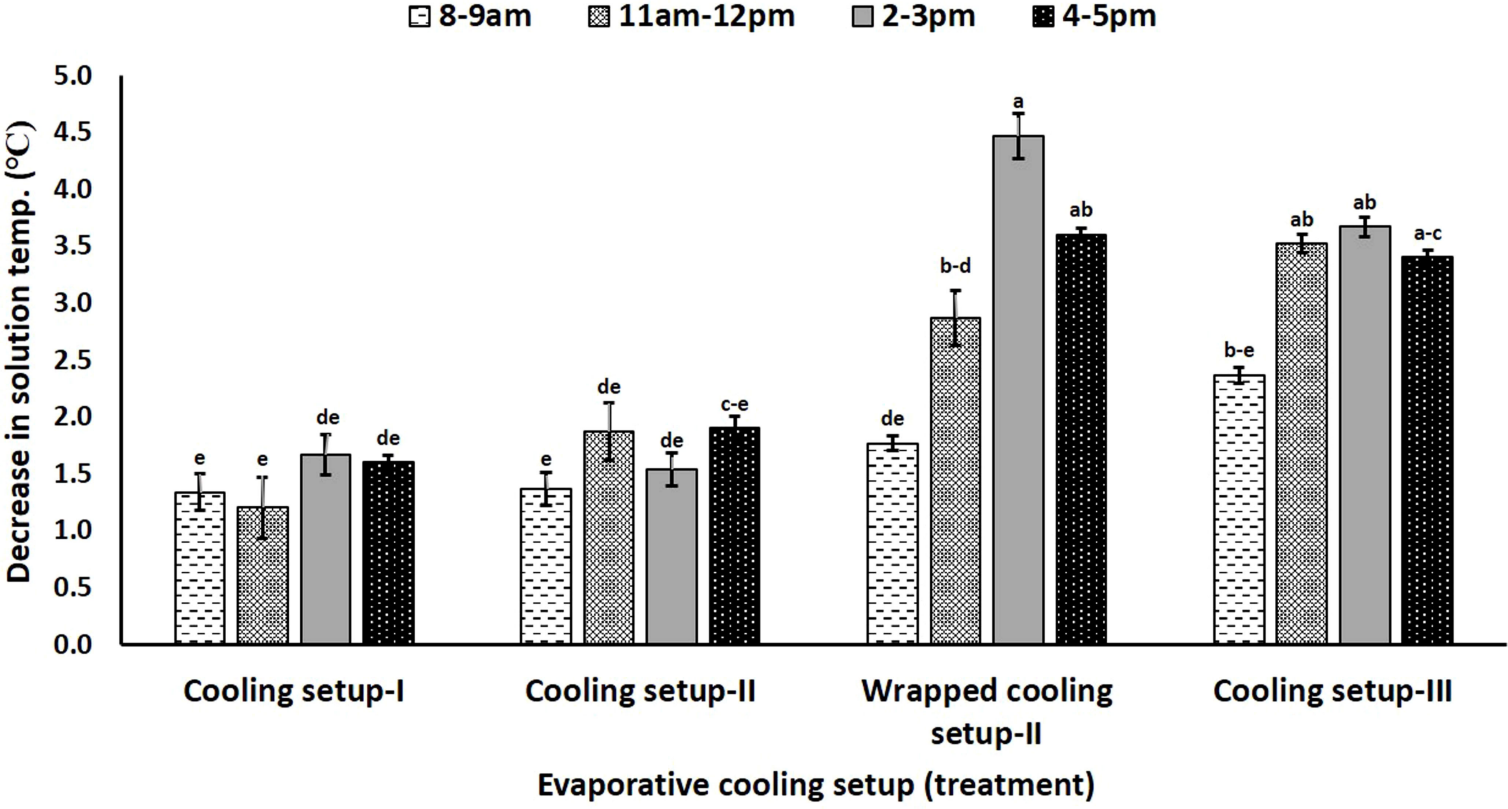
Impact of various non-electric evaporation techniques on decrease in nutrient solution temperature in nutrient film technique. Bars are means ± standard errors, and means sharing common letters do not differ significantly at p< 0.05.

The evaluation of the impact of cooling setups on nutrient solution temperature (p< 0.05) indicated decreases in nutrient solution temperature under all four cooling setups (**Figure 4**). The results demonstrated statistically similar decreases in nutrient solution temperature at all times of observations under Cooling Setup I and Cooling Setup II installments. A maximum of 1.8°C decrease in nutrient solution temperature was observed under Cooling Setup I and Cooling Setup II installments in comparison to control (no cooling setup installation). Wrapped Cooling Setup II and Cooling Setup III resulted in statistically similar reductions in solution temperature as compared to Cooling Setup I and Cooling Setup II from 8 a.m. to 9 a.m. (**Figure 4**). However, the temperature decreases under Wrapped Cooling Setup II were 168% and 191% higher than Cooling Setup I and Cooling Setup II, respectively, from 2 p.m. to 3 p.m. (**Supplementary Table 2**). To be precise, the temperature decreases due to Wrapped Cooling Setup II ranged from 3.6°C to 4.5°C and recorded as maximum from 2 p.m to 3 p.m. Cooling Setup III worked in a sustainable way from 11 a.m. to 5 p.m. and decreased nutrient solution temperature by approximately 3.5°C in comparison to control (**Figure 4**). Overall, the performances of Wrapped Cooling Setup II and Cooling Setup III to decrease the nutrient solution temperature were, statistically, alike (**Figure 4**).

### 3.3 Survival of summer vegetables in NFT hydroponic system having double-layer wet jute fabric-wrapped grow pipes attached with Wrapped Cooling Setup II

**Figure 5:**
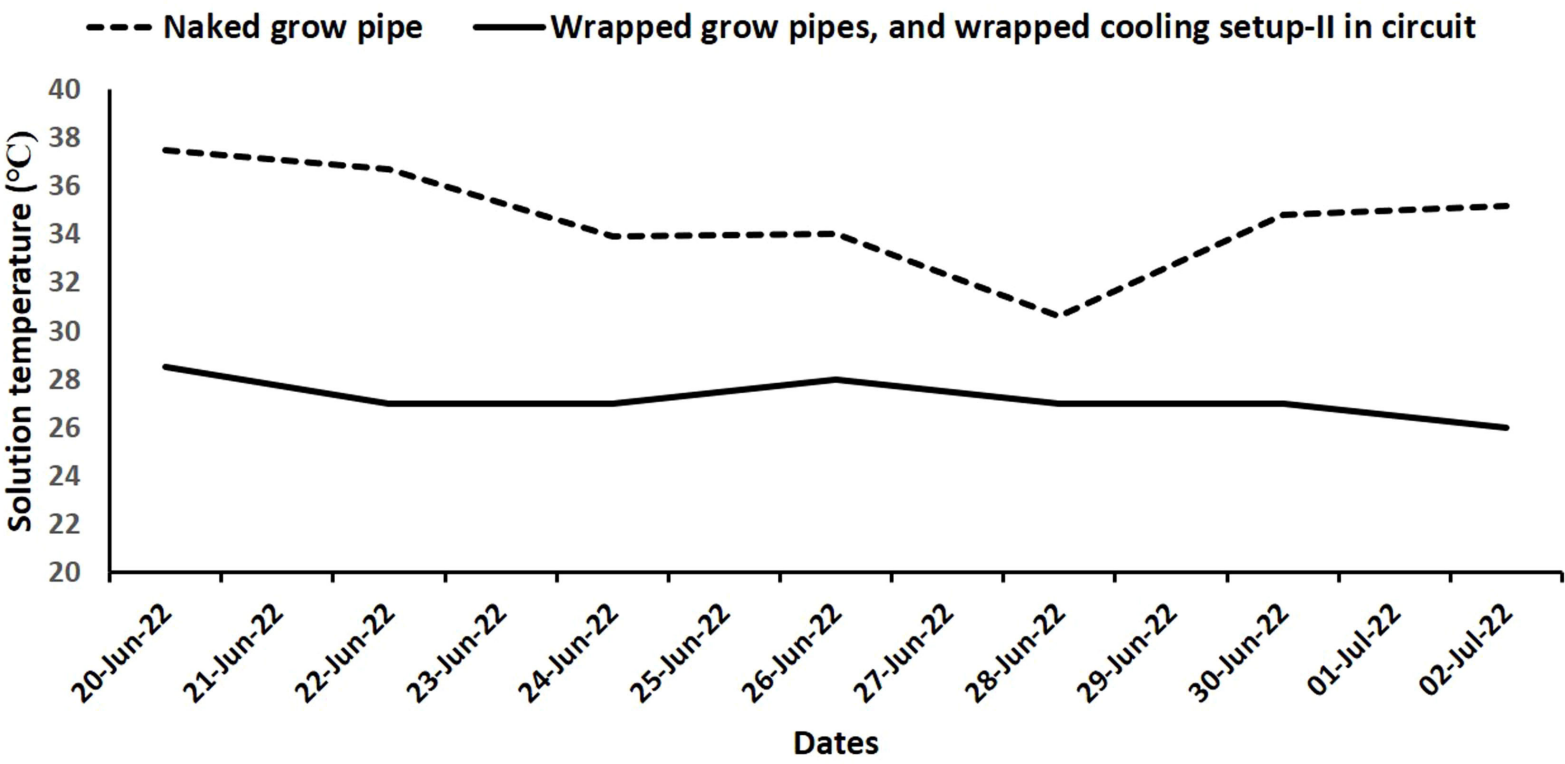
Comparison of nutrient solution temperature in naked grow pipes and wrapped grow pipes attached to wrapped Cooling Setup II during the first 2 weeks of test cultivation of vegetable crops.

The double-layer wet jute fabric-wrapped grow pipes attached with Wrapped Cooling Setup II reduced nutrient solution temperature by 3°C–8°C in comparison to control, i.e., naked grow pipes without any cooling setup installation (**Figure 5**). The temperature fluctuations in the nutrient solution of control remained between 30°C and 37°C, which resulted in vegetable seedling death within a week of the study period (**Table 1**). However, five out of 11 vegetable plants, viz., brinjal (*S. melongena*), bitter gourd (*M. charantia*), sponge gourd (*L. aegyptiaca*), bottle gourd (*L. siceraria*), and cucumber (*C. sativus*), survived in the double-layer wet jute fabric-wrapped grow pipes attached to Wrapped Cooling Setup II (**Tables 2**, **3**). Nonetheless, the wilting of the other seven vegetable plants took 2–26 days, a greater survival time in comparison to that noted in naked grow pipes (**Table 1**).

## 4 Discussion

An evaluation of very unique non-electric cooling techniques was carried out for their effectiveness in reducing the nutrient solution temperature of an NFT hydroponics system for the cultivation of summer vegetables. The nutrient solution temperature maintenance enables plant roots to uptake nutrients and water to ensure optimum growth and development (Solfjeld and Johnsen, 2006; Yan et al., 2012). In this study, the initial death of vegetable seedlings in naked grow pipes was the result of increased temperature (40°C) in nutrient solution. Summer vegetable canopies are supposed to withstand high temperatures, but their roots need to reside at below 30°C (86°F), which is maintained in the soil system (Suzuki, 1966). Thus, a temperature value higher than the optimum level would have seized the physico-chemical processes in roots, leading to the impairment of photosynthetic activity owing to a lack of sufficient water and nutrient supply (Xia et al., 2021). The minimum exposure to the external environment, through jute fabric wrapping of grow pipes, reduced the nutrient solution temperature and improved the photosynthetic framework of leaves. Given the differences in heat intensities of the external environment (Hooks et al., 2022), the decrease in nutrient solution temperature proved more effective during 11 a.m.–5 p.m. than that before 10 a.m. In addition to the reduced exposure to the external environment, these fluctuations in external temperature intensities were better regulated through wet jute fabric wrapping than dry jute fabric wrapping of grow pipes through the evaporative-cooling effect caused by the escape of high energy liquid molecules (Wang et al., 2017).

Given the principle of the “evaporative-cooling effect”, the installation of cooling setups with jute fabric-wrapped grow pipes proved successful in decreasing nutrient solution temperature. The various components of cooling setups, viz., porous walls of the bricks, earthen pot, and pitcher, allowed high-energy water molecules to escape the wet sand and evaporate. The attachment of double-layer wet jute fabric-wrapped grow pipes with wrapped Cooling Setup II and Cooling Setup III increased the effectiveness of the cooling systems in reducing nutrient solution temperature. We argued that the evaporative cooling of sand lowered the temperature of nutrient solution circulating in sand-buried aluminum coil in addition to the temperature decrease resulting from the evaporative cooling through double-layer wet jute fabric (Yang et al., 2019). Moreover, the wet jute fabric kept the evaporative surfaces wet and helped in establishing a continuity of evaporative streams, which, otherwise, might have broken due to the dryness of grow pipes, pitcher, or earthen pot surfaces. Additionally, more than evaporative cooling, the sunlight-exposed naked grow pipes experienced radiation energy-based direct heating (Guo et al., 2020). The absence of evaporative cooling-based temperature reductions made Cooling Setup I and Cooling Setup II less effective than Wrapped Cooling Setup II and Cooling Setup III.

The double-layer wet jute fabric-wrapped grow pipes attached to Wrapped Cooling Setup II maintained the nutrient solution temperature at 26°C–28°C, which was comparable to the recommended temperature of 26°C for hydroponics (González-García et al., 2023). This maintenance of nutrient solution temperature allowed five vegetable species (brinjal, bitter gourd, sponge gourd, bottle gourd, and cucumber) to survive till harvest. The other six vegetable species showed variable survival duration. This could be described as the plant-specific response to oxygen levels in the root zone. Plant species vary in their tolerance to suffocation under submergence or semi-submergence (Pradhan and Mohanty, 2013). Furthermore, the same nutrient solution recipe used for all vegetable species might have caused differential growth regulations in survived as well as wilted vegetable species. It was evident from the results of the experiment that the cooling setups never reduced nutrient solution temperature below air temperature during the morning time of respective days. Furthermore, a setup-induced decrease in temperature was less or negligible in the morning than that at noon or afternoon. These are the limitations of non-electric cooling setups studied, which provide future direction to explore the underlying reasons.

## 5 Conclusion

High temperature in nutrient solution is the major constraint for the survival of summer vegetable seedlings in an open-air NFT hydroponics system. The experimental results suggested that wrapping grow pipes with two layers of wet jute fabric can effectively reduce nutrient solution temperature through the evaporative-cooling effect. This effect can be further enhanced by introducing a non-electric cooling setup (Wrapped Cooling Setup II), which can keep the nutrient solution temperature within the plant tolerable range (26°C–28°C). The increased survival time of summer vegetables particularly brinjal (*S. melongena*), bitter gourd (*M. charantia*), sponge gourd (*L. aegyptiaca*), bottle gourd (*L. siceraria*), and cucumber (*C. sativus*) indicated that this method can be used to overcome the major constraint of high temperature in nutrient solution for the survival of summer vegetable seedlings in an open-air NFT hydroponics system. This method can be useful for farmers who want to grow summer vegetables in hot and dry climates.

## Data availability statement

The original contributions presented in the study are included in the article/**Supplementary Material**. Further inquiries can be directed to the corresponding authors.

## Author contributions

MM: Data curation, Investigation, Writing – original draft. RM: Conceptualization, Data curation, Formal Analysis, Methodology, Writing – original draft. ST: Data curation, Investigation, Writing – review & editing. MA: Data curation, Formal Analysis, Writing – review & editing. FN: Conceptualization, Methodology, Writing – original draft, Writing – review & editing. SB: Writing – review & editing. FW: Writing – review & editing. NA: Writing – review & editing. JL: Writing – review & editing. ZS: Writing – review & editing.
