# Corrigendum: Comparative efficacy of non-electric cooling techniques to reduce nutrient solution temperature for the sustainable cultivation of summer vegetables in open-air hydroponics

**DOI:** 10.3389/fpls.2024.1404645

**Published:** 2024-04-03

**Authors:** Muhammad Mohsin Nisar, Rashid Mahmood, Salman Tayyab, Moazzam Anees, Faisal Nadeem, Sadia Bibi, Faiza Waseem, Nazir Ahmed, Jing Li, Zhao Song

**Affiliations:** ^1^ Key Laboratory for New Technology Research of Vegetables, Vegetable Research Institute, Guangdong Academy of Agricultural Science, Guangzhou, China; ^2^ Department of Horticulture, University of the Punjab, Lahore, Pakistan; ^3^ Department of Soil Science, University of the Punjab, Lahore, Pakistan; ^4^ Institute of Soil and Environmental Sciences, University of Agriculture Faisalabad, Faisalabad, Pakistan; ^5^ College of Horticulture and Landscape Architecture, Zhongkai University of Agriculture and Engineering, Guangzhou, Guangdong, China

**Keywords:** hydroponics, nutrient solution temperature, PVC grow pipes, jute fabric, open air system

**Additional Affiliation(s)**

In the published article, there was an error regarding the affiliation(s) for “Muhammad Mohsin Nisar” and “Faisal Nadeem”. As well as having affiliation(s) “2” and “3” respectively they should also have “^1^Key Laboratory for New Technology Research of Vegetables, Vegetable Research Institute, Guangdong Academy of Agricultural Science, Guangzhou, China” as affiliation “1”.

As per above changes, the numbering of all author affiliations will be changed as mentioned above (correct version).

The authors apologize for this error and state that this does not change the scientific conclusions of the article in any way. The original article has been updated.

**Incorrect Author Name**

In the published article, an author name was incorrectly written as “Song Zhao”. The correct spelling is “Zhao Song”.

The initials used in the Author contributions section should be “ZS” instead of “SZ”.

The authors apologize for this error and state that this does not change the scientific conclusions of the article in any way. The original article has been updated.

