# Supplementary material for: Comparative efficacy of non-electric cooling techniques to reduce nutrient solution temperature for the sustainable cultivation of summer vegetables in open-air hydroponics
Source: Front Plant Sci. 2024 Mar 1;15:1340641. doi: 10.3389/fpls.2024.1340641 (PMC10940466; doi:10.3389/fpls.2024.1340641)
Supplement: Supplementary file 1 [file Table_1.docx]

| **Supplementary Table 1:** Percentage reduction in Temperature (℃) under different wrapping techniques | | | |
| --- | --- | --- | --- |
| **Description of Treatment** | **Single** | **Double** | **Percentage Change (%)** |
| **No. of Jute layers wrapped** | 4.12 | 4.93 | 19.66 |
| **Dry vs Wet Jute wrapping** | 1.87 | 5.28 | 182.35 |
| **Time of Observation** | 2.76 | 4.81 | 74.28 |

| **Supplementary Table 2:** Percentage Change in Temperature observed at different time periods under different cooling setups | | | | | | | | | | |
| --- | --- | --- | --- | --- | --- | --- | --- | --- | --- | --- |
| **Description** | **Cooling setup-I** | **Cooling setup-II** | **Wrapped cooling setup-II** | **Cooling setup-III** | **Percentage Change (%)** | | | | | |
|  |  |  |  |  | **With respect to Cooling setup-I** | | | **With respect to Cooling setup-II** | | **With respect to Wrapped Cooling setup-II** |
|  |  |  |  |  | **Cooling setup-II** | **Wrapped cooling setup-II** | **Cooling setup-III** | **Wrapped cooling setup-II** | **Cooling setup-III** | **Cooling setup-III** |
| **8-9am** | 1.3333 | 1.3667 | 1.7667 | 2.3667 | 2.51 | 32.51 | 77.51 | 29.27 | 73.17 | 33.96 |
| **11am-12pm** | 1.2 | 1.8667 | 2.8667 | 3.5167 | 55.56 | 138.89 | 193.06 | 53.57 | 88.39 | 22.67 |
| **2-3pm** | 1.6667 | 1.5333 | 4.4667 | 3.6667 | -8.00 | 168.00 | 120.00 | 191.31 | 139.14 | -17.91 |
| **4-5pm** | 1.6 | 1.9 | 3.6 | 3.4 | 18.75 | 125.00 | 112.50 | 89.47 | 78.95 | -5.56 |

| **Supplementary Table 3:** Percentage Change in Temperature observed in Wrapped grow pipes, and wrapped cooling setup-II in circuit with respect to Naked grow pipe | | | |
| --- | --- | --- | --- |
| **Description** | **Naked grow pipe** | **Wrapped grow pipes, and wrapped cooling setup-II in circuit** | **Percentage Change (%)** |
| **20-Jun-22** | 37.5 | 28.5 | -24.00 |
| **22-Jun-22** | 36.7 | 27 | -26.43 |
| **24-Jun-22** | 33.9 | 27 | -20.35 |
| **26-Jun-22** | 34 | 28 | -17.65 |
| **28-Jun-22** | 30.6 | 27 | -11.76 |
| **30-Jun-22** | 34.8 | 27 | -22.41 |
| **02-Jul-22** | 35.2 | 26 | -26.14 |
